# Measuring autism-associated traits in the general population: Factor structure and measurement invariance across sex and diagnosis status of the Social Communication Questionnaire

**DOI:** 10.1177/13623613231219306

**Published:** 2023-12-30

**Authors:** Laura Hegemann, Ragna Bugge Askeland, Stian Barbo Valand, Anne-Siri Øyen, Synnve Schjølberg, Vanessa H Bal, Somer L Bishop, Camilla Stoltenberg, Tilmann von Soest, Laurie J Hannigan, Alexandra Havdahl

**Affiliations:** 1 Department of Psychology, University of Oslo, Norway; 2 Nic Waals Institute, Lovisenberg Diaconal Hospital, Norway; 3 Norwegian Institute of Public Health, Norway; 4 Oslo University Hospital, Norway; 5 Graduate School of Applied and Professional Psychology, Rutgers University, USA; 6 Department of Psychiatry and Behavioral Sciences, University of California San Francisco, USA; 7 Department of Global Public health and Primary Care, University of Bergen, Norway; 8 MRC Integrative Epidemiology Unit (IEU), University of Bristol, UK

**Keywords:** cohort studies, factor analysis, MBRN, measurement invariance, MoBa, psychometrics, statistical, surveys and questionnaires

## Abstract

Autism screening questionnaires are sometimes used as a measure of “autism-associated traits” in samples drawn from the general population, even though such tools are primarily developed and designed for use in samples of children diagnosed with or being assessed for autism. Here, we explore the psychometric properties of the Social Communication Questionnaire (SCQ) current version reported at age 8 in a large population-based sample. Using data from the Norwegian Mother, Father and Child Cohort study (MoBa), we perform exploratory (*N* = 21,775) and confirmatory (*N* = 21,674) factor analyses on items and compare our results with previously suggested factor structure models of the SCQ. Furthermore, we test for measurement invariance across sex and registry-ascertained autism diagnostic status (*N*_diagnosed_ = 636). A 5-factor model provided best fit to the data in both children with and without autism diagnoses, though with some qualitative differences in what the factors represent across these groups. This model performed largely consistently across boys and girls in the general population. Taken together, the SCQ’s measurement properties must be carefully considered when it is used in population-based samples and measurement invariance testing of other autism screening tools used in similar contexts is warranted.

**Lay abstract:**

Using questionnaires in research relies on the expectation that they measure the same things across different groups of individuals. If this is not true, then interpretations of results can be misleading when researchers compare responses across different groups of individuals or use in it a group that differs from that in which the questionnaire was developed. For the questionnaire we investigated, the Social Communication Questionnaire (SCQ), we found that parents of boys and girls responded to questionnaire items in largely the same way but that the SCQ measured traits and behaviors slightly differently depending on whether the children had autism. Based on these results, we concluded that researchers using this questionnaire should carefully consider these differences when deciding how to interpret findings. SCQ scores as a reflection of “autism-associated traits” in samples that are mostly or entirely made up of individuals without an autism diagnosis may be misleading and we encourage a more precise interpretation of scores as a broader indication of social-communicative and behavioral traits.

## Introduction

Social communication difficulties and restricted, repetitive patterns of behavior or interests (RRBI) are the core diagnostic criteria for an autism spectrum diagnosis (hereafter autism) but are also multidimensional areas of development and behavior with relevance across the wider population. One group in which higher rates of these traits are typically seen is in non-autistic family members of those with a diagnosis (Frazier et al., 2015; Piven et al., 1997). This observation has had broad implications for etiological understandings of autism, including contributing to dimensional views of autism. However, social communication difficulties and RRBI are non-specific to autism (Lord & Bishop, 2021). Deriving insights about autism from studies of these traits in non-autistic or predominantly non-autistic samples therefore relies on the assumption that questionnaire-based screening instruments primarily developed and tested in samples of individuals with an autism diagnosis measure the same thing in population-based and largely non-autistic samples. Verifying this assumption—known as “measurement invariance”—is necessary to clarify how information from population-based studies of social communication difficulties and RRBI can be interpreted and used.

There are several questionnaire-based screening instruments for autism, measuring a range of social communication difficulties and RRBI, and developed for use in different contexts and ages. The Social Communication Questionnaire (SCQ; Rutter et al., 2003), designed to parallel the Autism Diagnostic Interview–Revised (Lord et al., 1994), is a parental report screener for children aged 4 years and older. The questionnaire covers three domains reflecting *Diagnostic and Statistical Manual of Mental Disorders* (4th ed.; *DSM*-IV) criteria of social interaction, communication, and RRBI. The SCQ was originally intended either for use as a second-level screening instrument (i.e. to be administered after initial concerns about development have been raised) or as a comparative measure of signs of autism between clinical groups or over time (Rutter et al., 2003). However, the SCQ, as well as other autism questionnaires, are sometimes used in general population or population-based samples and other largely non-autistic samples for a range of different applications, including as a quantitative measure of “autism-associated traits” (Bjørk et al., 2018; Firestein et al., 2022; Kim et al., 2021; Sasson & Bottema-Beutel, 2022).

There is substantial literature on the screening accuracy of the SCQ for identifying children with autism. Most of these studies are in clinical samples, but there are some in population-based samples (Chesnut et al., 2017; Surén, Saasen-Havdahl, et al., 2019) and a previous study has examined distributions of SCQ-scores in the general population (Mulligan et al., 2009). However, there is limited work assessing what trait dimensions or factors are measured by the SCQ in the general population and whether these factors differ or are measured differently between those with and without autism. The current version of the questionnaire as opposed to the life-time version may be more relevant in analyses using the SCQ as a measure of continuous traits given it measures behaviors present at the time of administration. Therefore, investigating the psychometric proprieties of the SCQ current version could be informative to the appropriate interpretation and use of the instrument as an “autism-associated trait” measure in population-based samples.

The factor structure of the SCQ, for both the life-time and the current versions of the instrument, has been investigated in primarily clinical samples (Berument et al., 1999; Gau et al., 2011; Grove et al., 2019; Karaminis & Stavrakaki, 2022; Kidd et al., 2020; Liu et al., 2022; Magyar et al., 2012; Martin et al., 2014; Wei et al., 2015) and in several samples when combined with other autism or attention-deficit hyperactivity disorder (ADHD) questionnaires (Krakowski et al., 2022; Warrier et al., 2022). Factor analysis aims to explain the correlational structure among items in a scale by modeling latent factors that are thought to influence individuals’ responses on the items. Factor analyses of the SCQ have typically tested the fits of models based on clinical criteria or ascertained in a clinical cohort. Commonly these studies include either or both a 2-factor model based on the *Diagnostic and Statistical Manual of Mental Disorders* (5th ed.; *DSM*-5) criteria for autism (social communication and RRBI), a three-factor model based on *DSM*-IV criteria (social interaction, communication, and RRBI) and/or the Berument et al. (1999) solution containing factors covering social behavior, communication, atypical language, and stereotyped behavior. Less is known about the factor structure in general population samples, and prior work has relied on using models based on theories developed in autism samples, such as diagnostic criteria (Evans et al., 2019), and theorizing them to be translatable to general population samples.

In addition to questions surrounding measurement consistency across diagnosis status, it is also necessary to consider potential sex differences in the properties of autism instruments. Understanding whether and how the SCQ performs differentially across sex is important as the underpinnings of sex differences in autism is an active area of research. For example, the “female protective effect” hypothesis suggests that females require a higher magnitude of predisposing factors (usually genetic liability) to be diagnosed with autism compared to males (Robinson et al., 2013; Wigdor et al., 2022). In addition, diagnostic considerations reflecting biases due to less recognition of differences in female presentations of autism or higher compensatory behaviors (Lai et al., 2015, 2017; Livingston & Happé, 2017) may lead to sex differences in the psychometric properties of autism instruments. The implications of these explanations differ markedly, so research to help disentangle them is vital. Data from instruments such as the SCQ applied in the general population may be able to contribute to this research, but understanding how their measurement differs (or not) across sex is necessary to make valid inferences.

A few studies have addressed sex differences of the SCQ in primarily autistic samples (Evans et al., 2019; Karaminis & Stavrakaki, 2022; Krakowski et al., 2022; Wei et al., 2015). These studies find some differences in specific items’ psychometric properties such as item functioning or how often items are endorsed. Krakowski et al. (2022) tested formally for measurement invariance; however, in these analyses, the SCQ was combined with an ADHD measure. Sex differences in the performance of the SCQ, and other instruments like it, may be difficult to detect in clinical samples as these samples are more likely to be predominantly male and might already reflect a sex bias in selection and ascertainment. Population-based samples in which parents enroll on behalf of their children do not usually suffer from this kind of ascertainment bias relating to the sex of the child.

In the present study, we take advantage of a large-scale population-based sample, the Norwegian Mother, Father, and Child Cohort Study (MoBa) cohort, with linkage to health registry data containing information about autism diagnoses to: (1) take a hypothesis-free approach to examine the underlying structure of the SCQ current version in a population-based sample at age 8; (2) compare this structure to factor structures derived from clinically ascertained samples or based on diagnostic criteria; and (3) assess assumptions of measurement invariance across both sex and autism diagnostic status.

## Methods

### Sample

The Norwegian Mother, Father and Child Cohort Study (MoBa) is a prospective population-based pregnancy cohort study conducted by the Norwegian Institute of Public Health (Magnus et al., 2006, 2016). Participants were recruited from all over Norway from 1999 to 2008. The women consented to participation in 41% of the pregnancies. The cohort includes approximately 114,500 children, 95,200 mothers, and 75,200 fathers. The current study is based on version 12 of the quality-assured data files released for research in January 2019. The establishment of MoBa and initial data collection was based on a license from the Norwegian Data Protection Agency and approval from The Regional Committees for Medical and Health Research Ethics. The MoBa cohort is currently regulated by the Norwegian Health Registry Act. The current study was approved by The Regional Committees for Medical and Health Research Ethics (2016/1702).

We use data from all MoBa children for whom mothers had completed the SCQ (Rutter et al., 2003) when their children were 8 years old (*N* = 43,449; *M_age_* = 8.18, *SD* = 0.18 years). Children’s sex was ascertained from the Medical Birth Registry of Norway (MBRN) so that sex refers to sex assigned at birth rather than gender. In total, 51% of the children in the sample were male and 49% female. Highly educated and older mothers are somewhat overrepresented in MoBa. Furthermore, participants with lower education levels were lost to follow-up at higher rates than those with higher education levels, increasing the overrepresentation of highly educated mothers in the present sample (Biele et al., 2019; Vejrup et al., 2021). Mothers of children with an autism diagnosis had lower completion rates of the 8-year questionnaire than mothers without (~5% difference) but similar rates of missingness across items within the SCQ. Children who were reported to not have phrase speech at the time of completing the questionnaire were a small minority in our sample, only making up 2.8% (*N* *=* 18) of those with an autism diagnosis and 0.7% (*N* *=* 294) of those without. Participants were not asked to report ethnicity.

### Measures

We included 38 of the 40 dichotomous (yes/no) items from the current version of the SCQ. Item 1, “Is your child able to talk using short phrases or sentences,” was excluded from factor analyses because it is not included in the scoring but only intended to assess whether the verbal items (Items 2–7) are applicable. Those who answered “No” to Item 1 were set to missing for those items. We also excluded Item 25, “does your child shake his/her head to indicate no,” as it was almost perfectly correlated with Item 24 “does your child nod his/her head to indicate yes.”

Clinical diagnoses of autism in MoBa participants were captured by the Norwegian Patient Registry (NPR) between 2008 and June 2021. The subsample of individuals with a recorded autism diagnosis included International Classification of Diseases, 10th Revision (ICD-10) codes for F84.0 (Childhood Autism), F84.1 (Atypical Autism), F84.5 (Asperger syndrome), F84.8 (Other pervasive developmental disorder), and F84.9 (Pervasive developmental disorder unspecified).

### Exploratory and confirmatory factor analyses

We conducted exploratory factor analyses (EFA) in one randomly selected half of the sample (EFA-sample; *N* = 21,775). To determine the optimal number of factors to retain, we used a scree plot; results from parallel analysis, optimal coordinates, and acceleration factor; as well as compared fit indices (comparative fit index (CFI), Tucker–Lewis index (TLI), standardized root mean square residual (SRMR), root mean square error of approximation (RMSEA)) across models extracting 1–10 factors. Theoretical interpretability of the factors and parsimony of the model were also considered when selecting the best solution. Based on these metrics, models with reasonable performance were run using confirmatory factor analysis (CFA) using the other half of the sample (CFA-sample; *N* = 21,674). In these models, items were specified to only load onto their highest loading factor in the EFA. The final model was selected from these models balancing fit indices from the CFA with parsimony of the model. As a sensitivity analysis, we also ran the final model based on the EFA results with a method factor to account for negatively worded items. In the CFA sample, three alternative factor models based on previous literature were also run. The alternative models were: a 2-factor model with two factors covering the *DSM*-5 dimensions; a 3-factor model with the SCQ domains based on *DSM*-IV criteria; and the Berument et al. (1999) 4-factor solution.

### Measurement invariance testing

Prior to formal measurement invariance testing, four models (the final model derived from the EFA and the alternative models) were run in subsamples of our total sample based on sex (*N_males_* = 22,119, *N_females_* = 21,253) and autism diagnosis status (*N_nodx_* = 42,736, *N_dx_* = 636), with sensitivity analyses excluding individuals that were present in the sample used for the EFA. Measurement invariance testing was then conducted across both sex and autism diagnostic status by means of multiple group structural equation modeling, where groups were defined according to sex and having received a diagnosis of autism or not, respectively. We tested increasingly more restrictive assumptions^1^ on which parameters of the model can be assumed to be equal across groups. Specifically, we first tested for *configural invariance* by examining whether the factor structure of the SCQ could be replicated across groups. Second, we tested for equality of factor loadings and thresholds across groups. Finally, we tested for the most restrictive assumption by testing the equality of the residual variances across groups (Kline, 2016; Wu & Estabrook, 2016).

We used change in the CFI and McDonald’s Noncentrality Index (McNCI) for formal comparison across the models. In the autism diagnosis status models, to account for the highly unbalanced group sample sizes, we ran 100 iterations of the models randomly subsampled with replacement from the non-diagnosed group. We used an average of the change in fit indices across the models to make the comparisons (Yoon & Lai, 2018). To assess if measurement invariance was met (i.e. whether constraints on parameters across groups at each level were acceptable), we used both conservative criteria (CFI: −Δ0.002 and McNCI: −Δ0.008; Meade et al., 2008) as a lower bound and standard criterion (CFI: −Δ0.01 and McNCI: −Δ0.02; Cheung & Rensvold, 2002) as a upper bound to reject the hypothesis of invariance, with results falling between being considered inconclusive and interpreted alongside differences in parameter estimates. For full technical details of the measurement invariance testing, sensitivity models testing partial invariance, the selection of fit indices for model comparisons, and the subsampling approaches for the diagnostic status analysis, see the supplementary methods.

### Software and analytic code

EFA and CFA were conducted using version 8 of the statistical software *Mplus* (Muthén & Muthén, 1998–2017). Analyses to estimate the number of factors to extract and measurement invariance models were run using the *nFactors* (v2.4.1), *psych* (v2.1.9), *lavaan* (v0.6-14), and *semTools* (v0.5-6) packages in R version 4.1.2 (Jorgensen et al., 2022; Raiche & Magis, 2020; R Core Team, 2021; Revelle, 2021; Rosseel, 2012). Diagnostic data was ascertained using the MoBa *phenotools* (v2.9) package (Hannigan et al., 2023). See supplementary methods for further details.

Analytic code for the analyses is openly available at https://github.com/psychgen/autism-scq-meas-invariance. The consent given by the participants does not allow for storage of data on an individual level in repositories. Researchers can apply for access to data for replication purposes via MoBa, in line with MoBa data access policies.

### Community involvement statement

The general population of parents and youth in MoBa have been engaged in focus groups guiding the development of the MoBa questionnaires. The Norwegian national associations for autism have provided input on selection of measures to include in the MoBa questionnaires, including the SCQ. Discussions and ultimate decision to include the SCQ in MoBa were by the Autism Birth Cohort Study (Stoltenberg et al., 2010). The Lovisenberg Hospital User Panel provided input on the development of the grant application funding this work and discussion of results.

## Results

The mean total score for the SCQ was 3.22 (*SD* = 2.81) in the full sample and 3.65 (*SD* = 3.03) in boys, 2.76 (*SD* = 2.48) in girls, 7.99 (*SD* = 6.01) in those with an autism diagnosis, and 3.15 (*SD* = 2.67) in those without. Scores for subdomains (Table S1) and item prevalence rates (Tables S2–S6) in the full sample and across sex and diagnosis status alongside formal comparisons of score means and item prevalence across the groups (Tables S1, S7) are presented in the supplementary materials.

### Ascertaining the factor structure of the SCQ in a general population sample

#### Exploratory factor analyses

**Figure 1.**
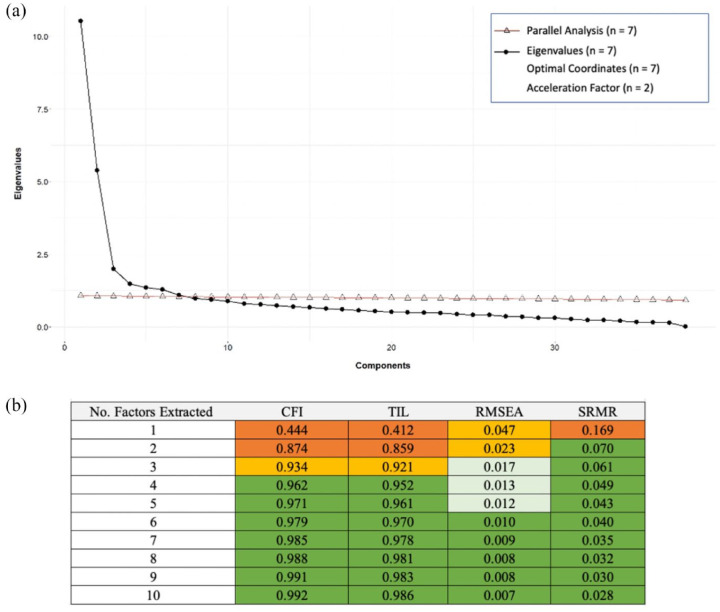
(a) Scree plot and results of the parallel analysis, eignvalues, optimal coordinates, and acceleration factor are present. (b) Fit indices for EFAs extracting 1–10 factors. Fit criteria; RMSEA: excellent < 0.01, good < 0.05, mediocre < 0.08, < 1 poor. CFI and TLI: excellent > 0.97, good > 0.95, acceptable > 0.9, poor < 0.9. SRMR: acceptable < 0.10, good < 0.08.

Results from EFA of the SCQ are presented in Figure 1. Statistical methods and interpretation of the scree plot (panel a) alongside fit indices (panel b) indicated solutions with 2–7 factors. Ultimately, the 5-factor solution was selected because it was a more parsimonious model than the 6- and 7-factor solutions and, compared to the 2- to 4-factor models, had better fits in both the EFA and CFA samples (EFA: panel b; CFA: Table 1) with more interpretable factors.

**Table 1.** Fit indices presented for models run in the CFA sample.

Model	Chi-square	CFI	TLI	RMSEA	SRMR
Models based on results from the EFA					
3-factor model	χ^2^(662) = 12,308.707	0.803	0.790	0.028	0.116
4-factor model	χ^2^(659) = 10,132.608	0.839	0.829	0.026	0.111
5-factor model	χ^2^(655) = 6437.532	0.902	0.895	0.020	0.087
5-factor model w/ method factor	χ^2^(640) = 13,169.286	0.894	0.884	0.021	0.081
Alternative models					
2-factor (*DSM*-5 criteria)	χ^2^(593) = 16,028.627	0.707	0.689	0.035	0.153
3-factor (SCQ Domains/*DSM*-IV)	χ^2^(557) = 22,724.532	0.612	0.586	0.042	0.156
4-factor (Berument et al., 1999)	χ^2^(659) = 15,324.240	0.751	0.735	0.032	0.143

CFA: confirmatory factor analysis; CFI: comparative fit index; TLI: Tucker–Lewis index; RMSEA: root mean square error of approximation; SRMR: standardized root mean square residual; EFA: exploratory factor analyses; *DSM*-5: *Diagnostic and Statistical Manual of Mental Disorders* (5th ed.); SCQ: Social Communication Questionnaire; *DSM*-IV: *Diagnostic and Statistical Manual of Mental Disorders* (4th ed.).

**Figure 2.**
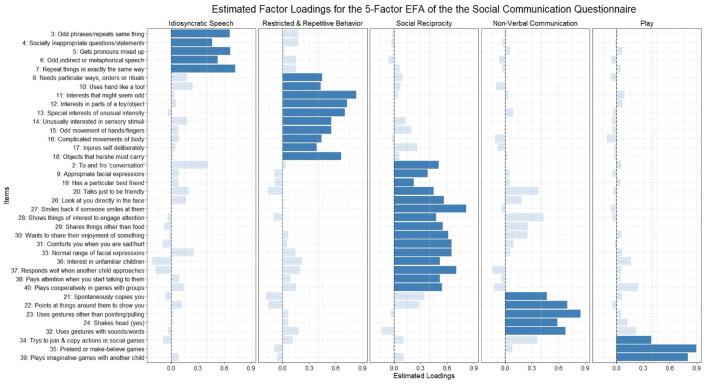
Estimated factor loadings from the 5-factor EFA. Darkened bars are items included in the CFA model of the 5-factor model. Dotted gray line shows a 0.4 factor loading.

The five identified factors in the EFA, indicated by the darkened bars in Figure 2, correspond to the domains of idiosyncratic speech (e.g. “repeat things in exactly the same way”), repetitive and restrictive behaviors or interests (e.g. “interests that might seem odd”), social reciprocity (e.g. “smiles back if someone smiles at them”), non-verbal communication (e.g. “uses gestures other than pointing/pulling”), and play (e.g. “plays imaginative games with another child”). Most items were fair indicators of their respective factors (λ > 0.4) with four exceptions (Items 9, 17, 19, and 34; see Figure 2). Only Items 2 and 28 had cross loadings greater than 0.4, both being under 0.45. Solutions with fewer factors had substantially more items with cross loadings greater than 0.4 and/or weaker estimated loadings on their primary factor than in the 5-factor model (Tables S8–S11).

#### Confirmatory factor analyses

In the CFA using the other half of the sample, the 5-factor model (Figure S1) as developed through the EFA fits the data adequately according to most fit indices, even though the TLI was somewhat low (Table 1). The addition of a question wording method factor decreased fit slightly for most indices. In this half of the sample, the items in the 5-factor model were good indicators of their respective factors as only two items had standardized factor loadings under 0.4 (Items 9 and 19). Factors were positively correlated with each other except for the “non-verbal communication” factor which was slightly negatively correlated with “restrictive, repetitive behaviors or interests” and “idiosyncratic speech” factors, and the “play” factor which was uncorrelated with the “idiosyncratic speech” factor.

We then compared the 5-factor model to alternative factor structures for the SCQ from the literature (Table 1). The Berument 4-factor model fit the data poorly but was the best out of the alternative models tested. The 2-factor *DSM*-5 model and the original 3-factor SCQ factor model also fit exceptionally poorly. Items 9 and 19, which were poor indicators in the 5-factor model, also had factor loadings under 0.4 for their specified factors in any of the alternative factor models. The idiosyncratic speech factor identified in the 5-factor solution was the same as the atypical language from the Berument 4-factor model. Other factors did not directly overlap with factors from other solutions, but the RRBI factor was very similar across the 5-factor solution, 3-factor *DSM*-IV model, and Berument model. The non-verbal communication and social reciprocity factors were closest to the social and communication factors in the 3-factor *DSM*-IV model.

### Testing measurement invariance across sex and diagnostic status

#### Sex

**Figure 3.**
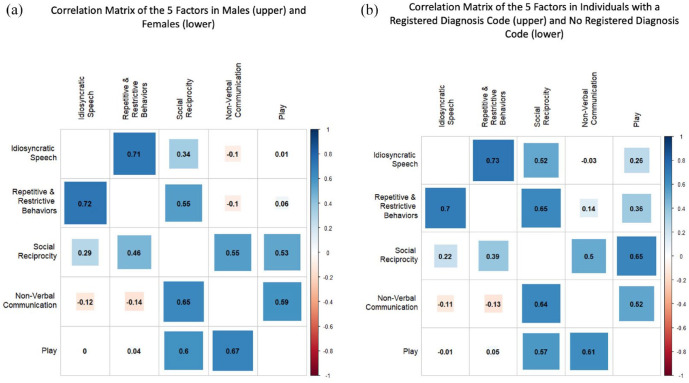
Correlations matrices of the 5-factor model when run in subsamples for (a) males (above the diagonal) and females (below the diagonal) and (b) those with a registered diagnosis code of autism in NPR (above) and without (below).

The 5-factor model provided an overall acceptable fit to the data when run separately in boys and girls, although CFI/TLI were slightly under an acceptable range in boys (Girls—CFI: 0.911, TLI: 0.905, RMSEA: 0.017, SRMR: 0.091; Boys—CFI: 0.891, TLI: 0.883, RMSEA: 0.022, SRMR: 0.083). This model was a better fit in both groups than the alternative models from the literature (Table S16). The estimated factor correlations from these models are shown in Figure 3, with girls below the diagonal and boys above. Factors were similarly correlated in both groups.

We found that both the configural invariance models had acceptable fits comparable to the model in the full sample (Table 2) and invariance of thresholds and loadings held, indicating the same factor structures for boys and girls, with factors measuring equivalent constructs. When assessing equivalent residual variances of the items, the invariance assumption was inconclusive. Further investigations revealed that the differences in fit indices could be attenuated by freeing the residual variance of Item 13 (“special interests of unusual intensity”), indicating that sex differences in measurement precision could be largely attributed to this item.

**Table 2.** Measurement invariance testing across sex.

Model	Compared to	CFI	ΔCFI	McNCI	ΔMcNCI	TLI	RMSEA	Invariance holds
Sex								
Configural	NA	0.902		0.862		0.895	0.020	Yes
Loadings/Thresholds	Configural	0.906	0.004	0.859	–0.003	0.901	0.019	Yes
Residual variances	Loadings/Thresholds	0.901	–0.005	0.847	–0.012	0.899	0.019	Yes/No
Autism diagnostic status								
Configural	NA	0.914		0.838		0.907	0.019	Yes
Loadings/Thresholds	Configural	0.909	–0.005	0.816	–0.022	0.904	0.020	Yes/No
Residual variances	Loadings/Thresholds	0.905	–0.003	0.789	–0.027	0.903	0.020	Yes/No

CFI: comparative fit index; McNCI: McDonald’s Noncentrality Index; TLI: Tucker–Lewis index; RMSEA: root mean square error of approximation; NA: not applicable.

Conservative Criteria; ΔCFI = –0.002, ΔMcNCI = −0.008. Standard criteria: ΔCFI = –0.01, ΔMcNCI = –0.02. Yes/No refers to being under the standard criteria for either one or both ΔCFI and ΔMcNCI but exceeding the conservative criteria in both. Fit indices for the autism diagnostic status models represent the averaged indices over 100 iterations of the models, 99 of which converged.

#### Diagnostic status

The 5-factor model fit well when run separately in those with and without a registered autism diagnosis (Dx—CFI: 0.929, TLI: 0.924, RMSEA: 0.031, SRMR: 0.095; NoDx—CFI: 0.914, TLI: 0.907, RMSEA: 0.019, SRMR: 0.084) even when excluding individuals included in the sample for the EFA to guard against over-fitting (Table S16). The 5-factor model had the best fit compared with the alternative models from the literature in both groups, although the alternative models had relatively better fits in the diagnosis group than in the no diagnosis group (Table S16). The factor correlations from the 5-factor model in the two groups are shown in Figure 3, with the no diagnosis group below the diagonal and the diagnosis group above. The estimated correlations differed slightly across diagnostic status as, broadly speaking, the factors were more correlated in the diagnosed group than in the no diagnosis group. Particularly, the “repetitive and restrictive behaviors” and, to a lesser extent, the “idiosyncratic speech” factors were more correlated with factors covering social and communication traits in the diagnosed group.

**Figure 4.**
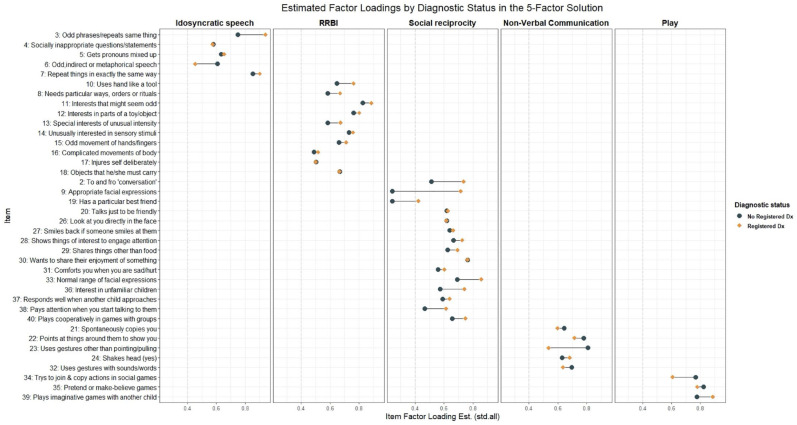
Differences in standard estimated factor loadings on the five factors shown for both those with a registered diagnosis and those without. Estimates from the configural invariance model.

The configural model across diagnosis status had an adequate fit (Table 2). Invariance of the thresholds and loadings could not be conclusively confirmed or rejected, as ΔCFI fell in-between our conservative and standard criteria we set to conclusively reject the hypothesis of invariance while ΔMcNCI exceeded both criteria. Examining parameter estimates to gain insight on the why change in both fit indices exceed our conservative fit criteria, we note that estimated loadings (Figure 4) and thresholds for some items between groups differed in the configural model. Some of these differences were sizable; for example, one loading showed a 66% decrease in magnitude from the diagnosed to the no diagnosis group. For comparison, between males and females—where fit indices conclusively supported the invariance of thresholds and loadings—the largest percent difference in loading between groups was 22%. As invariance of the thresholds and loadings held based on standard ΔCFI criteria, the invariance model testing equivalence of the residual variances was run but invariance assumption failed to hold based on ΔMcNCI and was inconclusive based on ΔCFI. Due to the invariance of thresholds and loadings not holding based on ΔMcNCI, we did not perform significance testing on differences seen in the factor correlations as the factors may represent different constructs and thus not directly comparable. Altogether, results indicated that the factor structure was the same across the two groups, but the factors may represent somewhat different constructs in those with and without an autism diagnosis.

## Discussion

A 5-factor model best accounted for the observed patterns of relationships among the SCQ items rather than solutions with fewer factors, suggesting higher dimensionality underlying these traits in a population-based cohort than, for example, two factors of social/communication and RRBI that may be assumed based on the current diagnostic criteria for autism. This model was the best fitting model in both boys/girls and those with and without an autism diagnosis. Formal measurement invariance testing supported similar factor structures across both sex and autism diagnosis status as well. More restrictive measurement invariance tests, however, suggested that while factors represented the same constructs between males and females, the nature of what the identified factors measure may differ between those with and without autism diagnoses.

### Factor structure underlying the SCQ in the general population

These results provide an important reminder that the factor structure of autism screening instruments in population-based samples cannot be assumed to be the same as those derived from clinical populations—especially when such samples clearly differ from those used in the development or testing of the instrument. Our results indicate that the SCQ measures social, communication, and RRBI traits in a population-based cohort across five dimensions. This is notably more than the 2–3 domains based on *DSM*-IV or *DSM*-5 criteria that is typically seen. The 5-factor model performed markedly better in our population-based sample than the alternative factor structures based on theory developed from autism clinical samples. This is consistent with a similar finding of increased heterogenicity underlying another frequently used autism screening instrument in the general population (Taylor et al., 2020). While the 5-factor model also provided the best fit to the data in the subsample of individuals with an autism diagnosis, the 2- to 3-factor models previously identified in clinical samples were relatively better fits in this group compared to the full sample.

In our 5-factor solution, the RRBI factor, the social reciprocity factor, and the non-verbal communication factor were similar to the corresponding factors from the 3-factor model based on *DSM*-IV criteria. The additional factors, both with comparably fewer items loading onto them, we labeled idiosyncratic speech and play. While items in the idiosyncratic speech factor have separated from larger factors in previous EFA work in clinical samples, including in the Berument solution (Berument et al., 1999; Warrier et al., 2022), the items in the play factor have not. This may reflect that many things influence differences in play behaviors, leading to reduced correlations of the items with the other social factors. Therefore, we interpret this finding as evidence of more complex heterogeneity in play behaviors in the general population. Practically, this suggests that sum scores with these items, particularly in a population-based cohort, are likely capturing multiple constructs which should inform interpretation of associations with these scores used in this context.

We found poor loadings of several more items, most covering social and communication traits, indicating some further heterogeneity influencing responses factor. Item 19 (“has a particular best friend”) did not have strong loadings on any factor—either in our 5-factor model in both the EFA and CFA or in any of the alternative models. This was also the only item that was a universally poor indicator in our study. The item just barely crossed a 0.4 threshold in those with a diagnosis and was a poor indicator in both girls and boys. The other items with low loadings in either our general population EFA or CFA models (EFA: Items 9, 17, 34; CFA: Item 9) have been observed as good indicators in clinical populations (Berument et al., 1999; Grove et al., 2019) and performed well in many of the models in our diagnosed subsample.

Besides the items with low loadings in the general sample, there were several other observations from our results that may suggest further heterogeneity being captured by the SCQ items in the general population: (1) many items had significant, although generally small, cross loadings across multiple factors in the EFA indicating items are measuring multiple constructs; (2) none of the models tested, either developed from our EFA or based on our literature, had more than just “acceptable” fits using standard CFI, TLI, and SRMR criteria in the general population; and (3) several methods to determine the number of factors indicated even more underlying factors. These observations may also highlight difficulties in using the SCQ as “trait” measure of underlying factors given its binary response options and intended use as a screener. Taken together, we suggest sum scores of the SCQ, particularly total scores or subscores based on *DSM*-5 diagnostic criteria, are likely capturing multiple factors. This supports arguments against using a total score for distinguishable and uncorrelated characteristics which would lack the coherent meaning implied by the term “autism traits” (Bottema-Beutel et al., 2021). Therefore, we recommend against using a SCQ total score as a measure of autistic traits in future etiological work in population-based samples. Instead, we note examining subscale or even item-level differences may be informative depending on the empirical context.

### Measurement invariance across sex

We found no evidence of any major sex differences in the way the SCQ measures social communication and RRBI traits in the general population. This is somewhat surprising given the literature on differences in presentations of autism behaviors in girls (or even a female autism phenotype) and concerns that current instruments may not be adequately capturing these behaviors in girls (Hull et al., 2020; Mandy & Lai, 2017). In a population-based sample, the SCQ can be assumed to be measuring the same underlying factors across sexes. The strictest invariance model failed to meet conservative criteria, suggesting that there may be small differences in the precision of the instrument to measure the factors between males and females.

The potential differences in measurement precision seems to be accounted for by Item 13 (“special interests of unusual intensity”) as partial invariance of the residual variances holds when the parameter for the item was allowed to differ between groups. This concurs with previous studies that have found this item to have differing psychometric properties between sexes, such as significant differential item functioning between girls and boys in an item response theory framework (Wei et al., 2015) and higher in the psychometric property of difficulty in girls compared to boys (Karaminis & Stavrakaki, 2022). The changes in fit indices between the invariance models are small and using standard criteria would support invariance of the residual variance. This suggests that, overall, observed scores between boys and girls in a population-based sample can be directly compared. However, given agreement with the previous literature regarding sex differences in the psychometric properties of Item 13, we caution against interpretation of sex differences in endorsement of this item as *solely* due to differences in the underlying behavior.

### Measurement invariance across autism diagnostic status

The 5-factor model provided a good fit in both those with and without autism, and configural invariance testing indicated that the number of factors and items loading onto those factors can be assumed to be the same across the two groups in our sample. Differences between the two groups emerged, however, when we tried to constrain items to be of equal importance to their factors across groups. Based on formal measurement invariance testing, we failed to conclusively uphold or reject the invariance of thresholds and loadings as both alternative fit indices exceed our conservative criteria to reject invariance but ΔCFI did not exceed the standard criteria, making the formal comparison inconclusive. Considering qualitative observations, some items had noticeable differences in estimated standardized factor loadings between the two groups. In some cases, these differences were observed to be systematic across factors (e.g. consistently higher loadings for RRBI and social reciprocity items among the autism group). Taking together, we think there is enough uncertainty in the results to prevent us from concluding that the relationship between observed scores and the factors are definitely the same between the two groups. Thus, we recommend caution should be taken in interpreting identical observed scores across two individuals with and without an autism diagnosis as equality of the individuals on the underlying factor. Parental concern about autism has been shown to influence parental reporting on other autism measures (Havdahl et al., 2017) and likewise these findings may be reflecting differences in mothers’ interpretation of the items. For example, parents with children with autism, who are familiar with the behaviors intended to be measured by SCQ items, may interpret the items differently than parents of non-autistic children who may be thinking of different behaviors. This should be considered in the use of SCQ scores in general population samples, as it emphasizes that the SCQ is more appropriately used as a measure of social communication or RRBI rather than “autism-associated traits.”

### Limitations

There are several limitations that should be kept in mind in the interpretation of these results. First, despite our efforts to minimize over-fitting—for example, by splitting our sample in half for the EFA and CFA—all analyses were exploratory and data-driven. Moreover, the relatedness between participants in MoBa (both in terms of siblings and more structural relatedness owing to MoBa parents being related to other MoBa parents) meant that even splitting the sample at random did not completely remove dependency between the two halves. Notwithstanding familial relatedness between the two half samples, other similarities (due to them coming from the same study, and therefore having been ascertained in the same nonrandom way) also limit their independence and increase the risks of over-fitting. Taken together, this means that over-fitting likely made some contribution to our results. While over-fitting can result in spuriously complex models (Forster & Sober, 1994), it is highly unlikely to be solely responsible for the large differences in fit between our 5-factor model and the simpler alternative structures we tested in the general population. Beyond simply the scale of these differences, the solution we identify is theoretically coherent, with 4 out of the 5 identified factors similar to those seen in previous factor solutions, and the separation of an additional “play” factor reflecting diverse contributions to difficulties in play behaviors in a population-based sample.

Second, autism diagnoses were ascertained by ever receiving a diagnostic code for autism in the national public registry from the years of 2008–2021. As a result, there may be individuals in the “no diagnosis” group who received diagnoses prior to 2008/after 2021 or remain undiagnosed. Misdiagnosis in the autism group is also a possibility, although 93% of those receiving a diagnostic code once in our sample received it a second time. A record review of MoBa data conducted in previous work showed few coding errors and that most autism diagnoses were well documented in health records (Surén, Havdahl, et al., 2019). In addition, the autism diagnosis group may differ from other clinical cohorts due to selective participation among MoBa families. Relatively few in the group were reported to not have phrase speech or co-occurring intellectual disability and had an older average age of first recorded diagnosis (9.15 years for those with NPR data available from birth, meaning a portion were not diagnosed when mothers filled out the questionnaire). These factors likely reflect a group that differs in make-up from what is typically seen in clinically ascertained cohorts, potentially contributing to the better fit of the five-factor model in the autism subsample compared to the alternative models from the literature. Furthermore, measurement invariance testing based on heterogeneous aspects of autism such as language ability may be beneficial. Finally, due to the low number of autism diagnoses in females in our sample and issues with model estimation relating from this, we chose not to compare model structures and measurement invariance by diagnostic status *and* sex, limiting what we can conclude on sex differences in measurement *between* general and clinical populations.

## Conclusion

Autism questionnaire-based screening instruments, such as the SCQ, can be used as a measure of social communication and RRBI traits in general population samples, but their relevance to understanding the etiology of autism depends on their measurement properties in these samples. The evaluation of the factor structure in the present analyses suggests a more nuanced multidimensionality among these traits in population-based samples—more so than is implied by structures previously derived from data collected in clinically ascertained samples. While we find the instrument’s measurement to be largely invariant across sex, we find some evidence that the dimensions measured by the SCQ—while consistent in terms of structure—may differ qualitatively between autistic and non-autistic individuals. Taken together, we caution against assuming endorsement of SCQ items as autism-associated traits in population-based samples. Furthermore, in future research using SCQ scores in population-based samples, using more than just total scores may be informative. In addition, care must be taken not to extend conclusions to the etiology of autism specifically, but rather the etiology of social, communication, and RRBI traits more generally. Finally, our findings add support to the importance of looking at measurement properties of other autism screening instruments used as trait measures in population-based samples.

## Supplemental Material

sj-docx-1-aut-10.1177_13623613231219306 – Supplemental material for Measuring autism-associated traits in the general population: Factor structure and measurement invariance across sex and diagnosis status of the Social Communication Questionnaire Supplemental material, sj-docx-1-aut-10.1177_13623613231219306 for Measuring autism-associated traits in the general population: Factor structure and measurement invariance across sex and diagnosis status of the Social Communication Questionnaire by Laura Hegemann, Ragna Bugge Askeland, Stian Barbo Valand, Anne-Siri Øyen, Synnve Schjølberg, Vanessa H Bal, Somer L Bishop, Camilla Stoltenberg, Tilmann von Soest, Laurie J Hannigan and Alexandra Havdahl in Autism

sj-xlsx-2-aut-10.1177_13623613231219306 – Supplemental material for Measuring autism-associated traits in the general population: Factor structure and measurement invariance across sex and diagnosis status of the Social Communication Questionnaire Supplemental material, sj-xlsx-2-aut-10.1177_13623613231219306 for Measuring autism-associated traits in the general population: Factor structure and measurement invariance across sex and diagnosis status of the Social Communication Questionnaire by Laura Hegemann, Ragna Bugge Askeland, Stian Barbo Valand, Anne-Siri Øyen, Synnve Schjølberg, Vanessa H Bal, Somer L Bishop, Camilla Stoltenberg, Tilmann von Soest, Laurie J Hannigan and Alexandra Havdahl in Autism

## Notes

1. Due to Social Communication Questionnaire (SCQ) items having dichotomous response sets, there are some limits on which invariance assumptions that can be tested independently; we formally tested three assumptions following recommendations from Wu and Estabrook (2016) and therefore deviate from using the terms weak/metric, strong/scalar, and strict invariance in favor of invariance of the specific parameters we examine. See supplementary methods for further details.
