# Supplementary material for: Measuring autism-associated traits in the general population: Factor structure and measurement invariance across sex and diagnosis status of the Social Communication Questionnaire
Source: Autism. 2023 Dec 30;28(8):2105–19. doi: 10.1177/13623613231219306 (PMC11360277; doi:10.1177/13623613231219306)
Supplement: sj-docx-1-aut-10.1177_13623613231219306 – Supplemental material for Measuring autism-associated traits in the general population: Factor structure and measurement invariance across sex and diagnosis status of the Social Communication Questionnaire [file sj-docx-1-aut-10.1177_13623613231219306.docx]

**Supplementary methods**

*Item prevalence of SCQ items*

Item prevalence was calculated for the full sample as well as in females, males, those with an NPR autism diagnosis, and those without an NPR autism diagnosis. Statistically significance differences in prevalence rates were calculated using a two-sample proportion test performed in R using the *stats* (v.4.1.2) package.

*Total score creation for SCQ*

Total scores for the SCQ were a sum score of all items. Subdomain scores were sum scores based on the domains outlined in the SCQ manual - Social, communication, and restricted and repetitive behaviors and interest. For individuals with missing data, the sum score was divided by the number of items answered in the SCQ for the total score or in the domains for the subdomain scores and multiplied by the total number of items in the total score (38) or domains (15,12,9 respectively). This value rounded to the nearest whole number was presented as the total and subdomain scores for these individuals. A Wilcoxon test was performed in R using the *stats* (v.4.1.2) package to compare the statistical significance of differences in means across groups.

*Measurement invariance testing*

The measurement models were specified using the *semTools* (v0.5-5; Jorgensen et al., 2021) Jorgensen, 2021) *measEQ.syntax* function to generate the syntax for these models in accordance to Wu & Estabrook (2016). For the baseline model which we referred to as the configural model, we constrained latent variable and item response variables’ residual variances to 1 and item intercepts to 0 for model identification, meaning these parameters were equal across groups. Using terminology of Wu & Estabrook (2016), this was identified using condition 8. This results in 172 uniquely estimated parameters (1) in the multigroup model where *K*, the number of thresholds per item is 1; *m*, the number of common factors, is 5; *p,* the number of unique factors, is 38; and *G*, the number of groups, is 2.

$$G(2p+\frac{m\left( m-1 \right)}{2} )$$

(1)

For the loadings/thresholds invariance model, thresholds and loadings were held equal across groups and intercepts constrained to 0 but residual variances of the items were estimated so that the model was more restrictive than the baseline model but not nested. This was in accordance to proposition 11 of Wu & Estabrook and results in 144 uniquely estimated parameters (2).

$$G\left( p+\frac{m\left( m+3 \right)}{2} \right)+p-2m$$

(2)

Finally for the residual variances invariance model, thresholds, loadings, and residual variances were all held equal across groups and intercepts remained constrained to 0. This was the most restrictive model with 106 uniquely estimated parameters (3).

$$\frac{Gm\left( m+3 \right)}{2}+2p-2m$$

(3)

In the diagnostic status invariance models, there was a highly unbalanced sample size (N_dx_= 42,736, N_nodx_ = 636) between the groups. To avoid biasing results towards falsely accepting invariance as best possible, a subsampling approach was used running 100 iterations of the MI models randomly sampling with replacement from the non-diagnosed group, based on the smallest sample size that would reliably result in model convergence (N_nodx_ = 3,816, N_dx_:N_nodx_ = 1:6). The alternative fit indices were averaged across the iterations to assess measurement invariance (Yoon & Lai, 2018). A model with the full samples was also run to compare differences in parameter estimates.

Measurement invariance was assessed by considering alternative fit indices given χ2 is overly sensitive to deviations in large samples with significant results potentially picking up trivial differences and since some models were not nested. Comparative Fit Index (ΔCFI) and McDonald´s Noncentrality Index (ΔMcNCI) were used based on findings that these fit indices are less sensitive to sample size and generally nonredundant with other fit indices(Cheung & Rensvold, 2002; Meade et al., 2008). We used both conservative and standard criteria as there is some evidence showing fit indices are less sensitive to lack of invariance in large models (such as ours) as well as unclear guidelines for criteria when using dichotomous indicators and large samples (Putnick & Bornstein, 2016).

*Partial measurement invariance testing*

All partial invariance models for the sex invariance models were based on modification indices. Partial residual variances invariance was tested by freeing individual items’ residual variances based on modification indices from the residual variances invariance model. Only up to 5 items were tested using this method to keep the number of items freed under 15%. Several other models for the residual variances invariance model with constrained means were also run based on modification indices with several combinations of these item residuals and factor means being tested to investigate which items or factor means may be driving changes in fit indices between models. Given changes to the models were based off modification indices and the issues with goodness of fit criteria outline above, partial measurement invariance was post hoc and evaluated largely on relative magnitude of change between different models.

*Software information*

EFA and CFA were conducted using version 8 of the statistical software *Mplus* (Muthén & Muthén, 1998-2017). EFA and CFA were run using the WLSMV estimation method and delta parameterization. The EFA was run with geomin oblique rotation applied. Missing data for all models was handled using pairwise deletion, as it is the default for ordinal data in *Mplus*. The EFA and all CFA models were clustered on maternal ID to account for sibling relatedness. CFI, TLI, RMSEA reported use a scaled chi-square for robust estimators (Satorra, 2000).

Tetrachoric correlation matrices and the analyses to estimate the number of factors to extract were estimated using the *nFactors* (v2.4.1) and *psych* (v2.1.9) packages in R version 4.1.2 (R Core Team, 2021; Raiche & Magis, 2020; Revelle, 2021). All measurement invariance testing was carried out using the *lavaan* (v0.6-14) and *semTools* (v0.5-6) packages in R with the WLSMV estimation method, theta parameterization, and pairwise deletion for missing data (Jorgensen et al., 2022; Rosseel, 2012). The *Tidyverse* packages, *corrplot, readxl, haven,* and MoBa *Phenotools* were used for data ascertainment, importing, and tidying as well as figure creation (Hannigan, 2023; Wei & Simko, 2021; Wickham et al., 2019; Wickham & Bryan, 2021; Wickham & Miller, 2021).

**Supplementary Figure:**


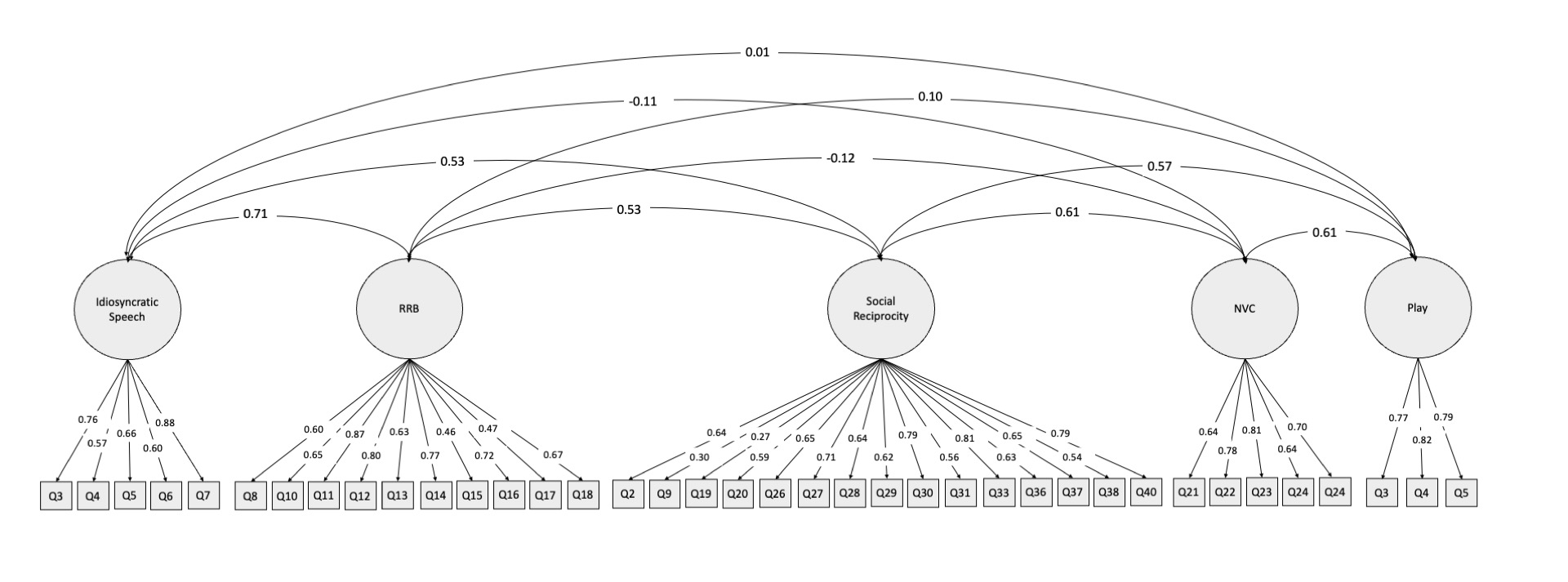


Figure S1: Path diagram for the 5-factor model developed from the EFA with std. factor loadings and factor correlations in the CFA-half sample.

**Supplementary References**

Cheung, G. W., & Rensvold, R. B. (2002). Evaluating Goodness-of-Fit Indexes for Testing Measurement Invariance. *Structural Equation Modeling: A Multidisciplinary Journal*, *9*(2), 233–255. https://doi.org/10.1207/S15328007SEM0902_5

Hannigan, L. (2023). *MoBa Phenotools* (0.2.9). https://github.com/psychgen/phenotools

Jorgensen, T. D., Pornprasertmanit, S., Schoemann, A. M., & Rosseel, Y. (2022). *semTools: Useful tools for structural equation modeling*. https://CRAN.R-project.org/package=semTools

Meade, A. W., Johnson, E. C., & Braddy, P. W. (2008). Power and sensitivity of alternative fit indices in tests of measurement invariance. *Journal of Applied Psychology*, *93*(3), 568–592. https://doi.org/10.1037/0021-9010.93.3.568

Muthén, L. K., & Muthén, B. O. (1998). *Mplus User’s Guide* (8th ed.). Muthén & Muthén.

Putnick, D. L., & Bornstein, M. H. (2016). Measurement Invariance Conventions and Reporting: The State of the Art and Future Directions for Psychological Research. *Developmental Review : DR*, *41*, 71–90. https://doi.org/10.1016/j.dr.2016.06.004

R Core Team. (2021). *R: A Language and Environment for Statistical Computing*. R Foundation for Statistical Computing. https://www.R-project.org/

Raiche, G., & Magis, D. (2020). *nFactors: Parallel Anaysis and Other Non Graphical Solutions to the Cattell Scree test* (R package version 2.4.1). https://CRAN.R-project.org/package=nFactors

Revelle, W. (2021). *psych: Procedures for Psychological, Psychometric, and Personality Research* (R package version 2.1.9). Northwestern University. https://CRAN.R-project.org/package=psych

Rosseel, Y. (2012). lavaan: An R Package for Structural Equation Modeling. *Journal of Statistical Software*, *48*(2). https://doi.org/10.18637/jss.v048.i02

Satorra, A. (2000). *Scaled and adjusted restricted tests in multi-sample analysis of moment structures*. Springer.

Wei, T., & Simko, V. (2021). *R package “corrplot”: Visualization of a Correlation Matrix* (Version 0.90). https://github.com/taiyun/corrplot

Wickham, H., Averick, M., Bryan, J., Chang, W., McGowan, L., François, R., Grolemund, G., Hayes, A., Henry, L., Hester, J., Kuhn, M., Pedersen, T., Miller, E., Bache, S., Müller, K., Ooms, J., Robinson, D., Seidel, D., Spinu, V., … Yutani, H. (2019). Welcome to the Tidyverse. *Journal of Open Source Software*, *4*(43), 1686. https://doi.org/10.21105/joss.01686

Wickham, H., & Bryan, J. (2021). *readxl: Read Excel Files* (R package version 1.3.1). https://CRAN.R-project.org/package=readxl

Wickham, H., & Miller, E. (2021). *haven: Import and Export “SPSS”, “STATA”, and ’SAS Files* (R package version 2.4.3). htpps://CRAN.R-project.org/package=haven

Wu, H., & Estabrook, R. (2016). Identification of Confirmatory Factor Analysis Models of Different Levels of Invariance for Ordered Categorical Outcomes. *Psychometrika*, *81*(4), 1014–1045. https://doi.org/10.1007/s11336-016-9506-0

Yoon, M., & Lai, M. H. C. (2018). Testing Factorial Invariance With Unbalanced Samples. *Structural Equation Modeling: A Multidisciplinary Journal*, *25*(2), 201–213. https://doi.org/10.1080/10705511.2017.1387859
